# Supplementary material for: Common Secondary and Tertiary Structural Features of Aptamer–Ligand Interaction Shared by RNA Aptamers with Different Primary Sequences
Source: Molecules. 2019 Dec 11;24(24):4535. doi: 10.3390/molecules24244535 (PMC6943582; doi:10.3390/molecules24244535)
Supplement: Supplementary file 1 [file molecules-24-04535-s001.pdf]

Supplemental Material

Methods

Simulation protocols

All molecular dynamics (MD) simulations were conducted using GROMACS 5.1.2 [1] with the Amber99sb all-atom force field [2]. The occupied aptamer coordinates were obtained from PDB entry 1NEM [3], fifth model, while unoccupied structure was constructed from the NMR structure by deleting the ligand. UCSD Chimera [4] and ACPYPE [5] a Python interface to Antechamber [6] were used to calculate the charge with AM1-BCC and prepare the neomycin ligand topology with the General Amber Force Field (GAFF) [7]. The aptamer was centered in a cubic box of TIP3P water molecules [8]. The distance between the aptamer and the box was 20Å. To neutralize the net charge of the aptamer, Na^+ ions were randomly placed as counterions in the system. Table S1 shows the number of counter ions, water molecules and the total number of atoms used in each simulation. Particle Mesh Ewald (PME) [9] was used for treating electrostatic interactions with grid-spacing of 1.6Å. The van der Waals interactions [10] were treated with a short-range cutoff of 1.0nm. Simulations were run for NEO1A aptamer neomycin occupied and unoccupied states.

Energy minimization was conducted via the steepest descent method [11]. The minimized structure was equilibrated using molecular dynamics with the NVT and NPT ensembles, respectively. The NVT thermal equilibration was carried out by velocity-rescaling temperature coupling [12] for 100ps at 298K. The NPT equilibration was conducted with Parrinello-Rahman pressure coupling [13] and the same velocity-rescaling temperature coupling. During equilibration, position restraints were applied to non-hydrogen atoms of the aptamer. The LINCS algorithm [14] was used to implement bond length constraints. The time step used was 2fs and periodic boundary conditions were applied to the system. Finally, an MD production simulation was carried out for 20ns at constant temperature (298K) and pressure (1.0bar) with the

aptamer, counterions and solvent molecules independently coupled to external heat baths with a relaxation time of 0.1ps. System coordinates were saved from the trajectory at 2ps intervals.

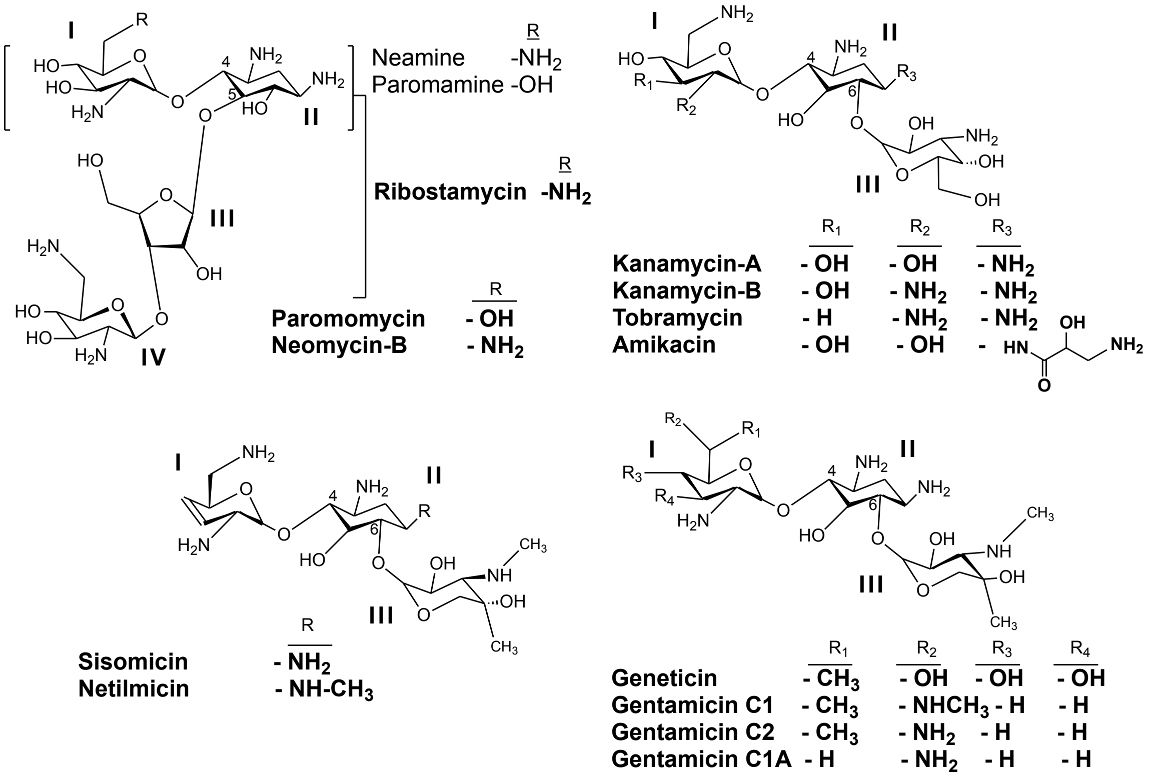

Figure S1. Aminoglycoside structures

The neomycin-class aminoglycoside antibiotics have a 2-deoxystreptamine (2-DOS, ring II), disubstituted at positions 4 and 5 positions while the kanamycin-class has substitutions at positions 4 and 6. The substitutions at the R positions are indicated for neomycin-class aminoglycoides. For kanamycin-class aminoglycosides, the main substations (R1 and R2) are located on the ring I. Sisomicin and netilmicin differ with only a single substitution at the R position on 2-DOS (ring II). The structure of geneticin differ from the rest of the aminoglycosides such that it has an extra methyl attached to C6 on the ring I, and the other two attached to ring III one on the amino at 3rd position and the other at 4th position. Gentamicin structure is similar to geneticin and the varying functional groups are indicated in their structures as R1 through R4.

Figure S2. Aminoglycoside binding to NEO2A determined by ITC

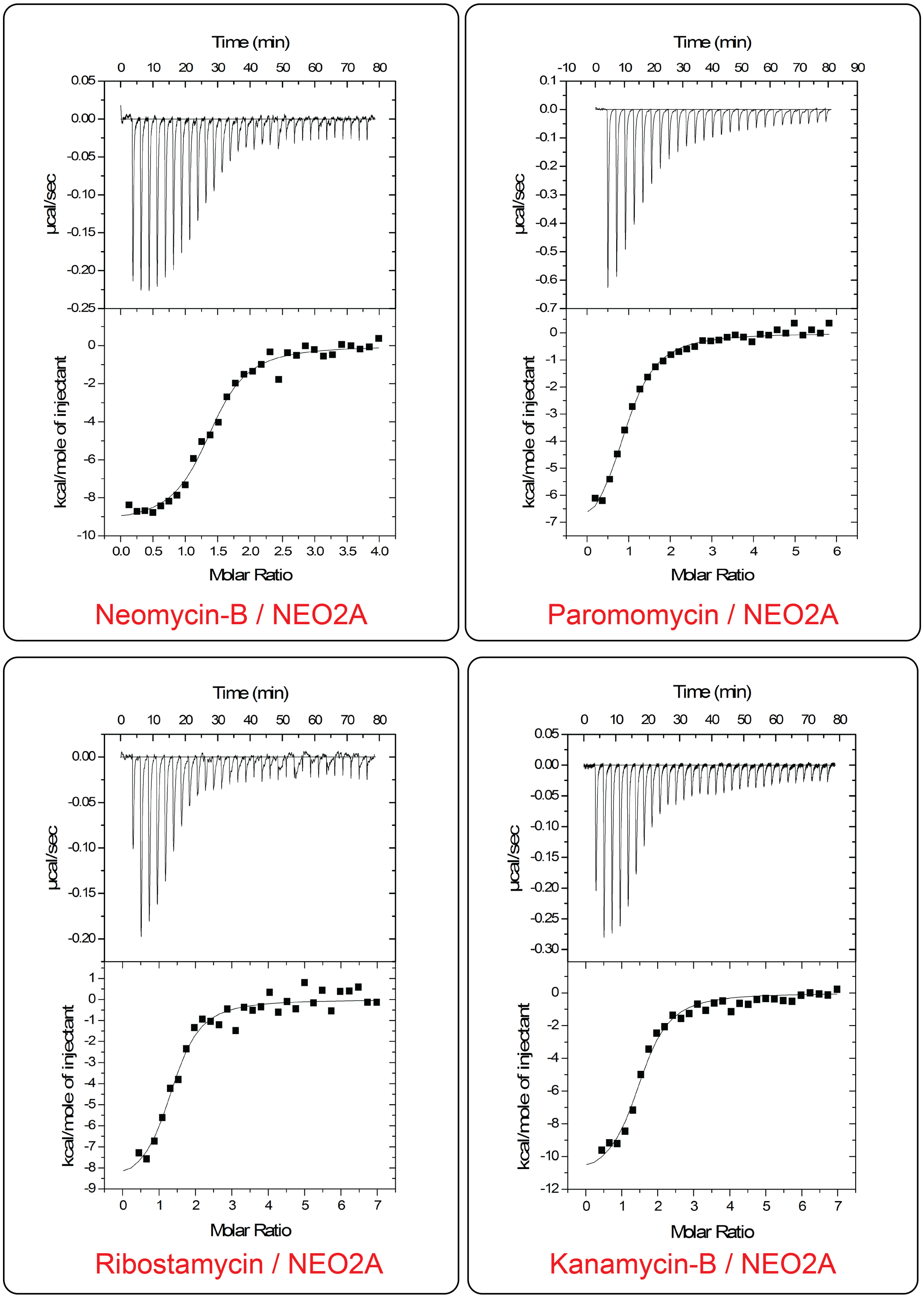

Representative NEO2A ITC binding data for different ligands as labeled.

Figure S3. Aminoglycoside binding to NEO2A determined by ITC

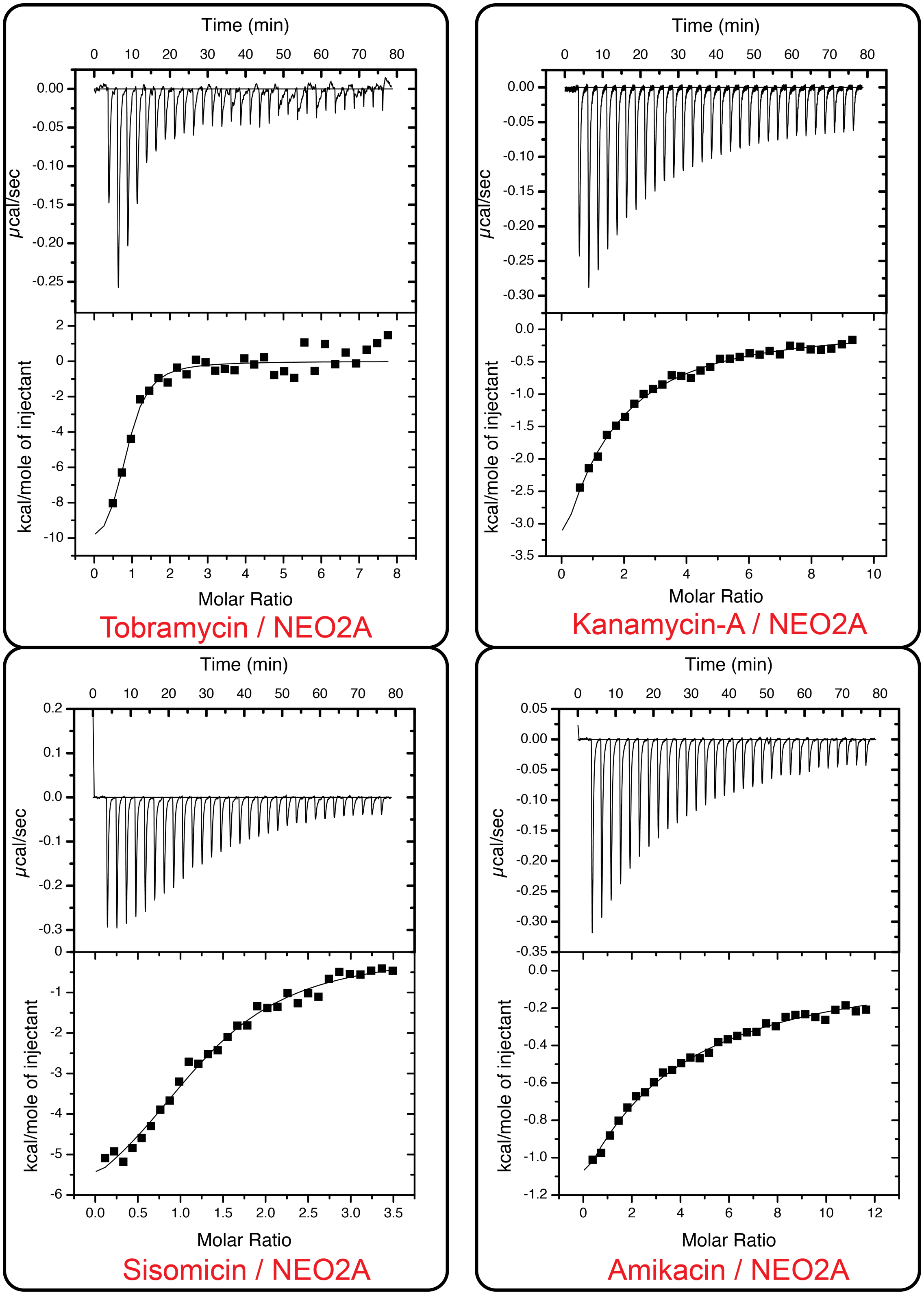

Representative NEO2A ITC binding data for different ligands as labeled.

Figure S4. Aminoglycoside binding to NEO2A determined by ITC

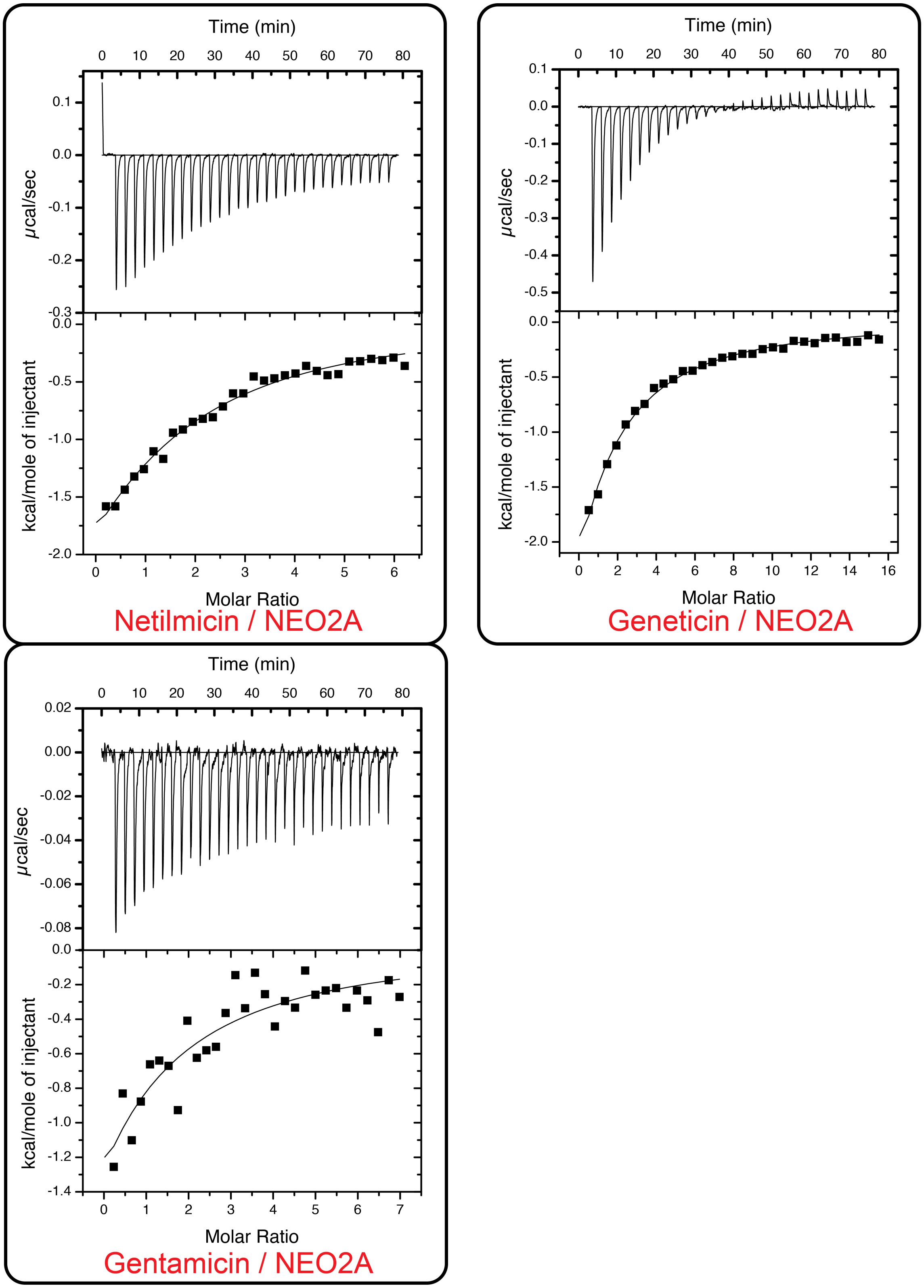

Representative NEO2A ITC binding data for different ligands as labeled.

Figure S5. 2AP fluorescence in the presence and absence of ligands

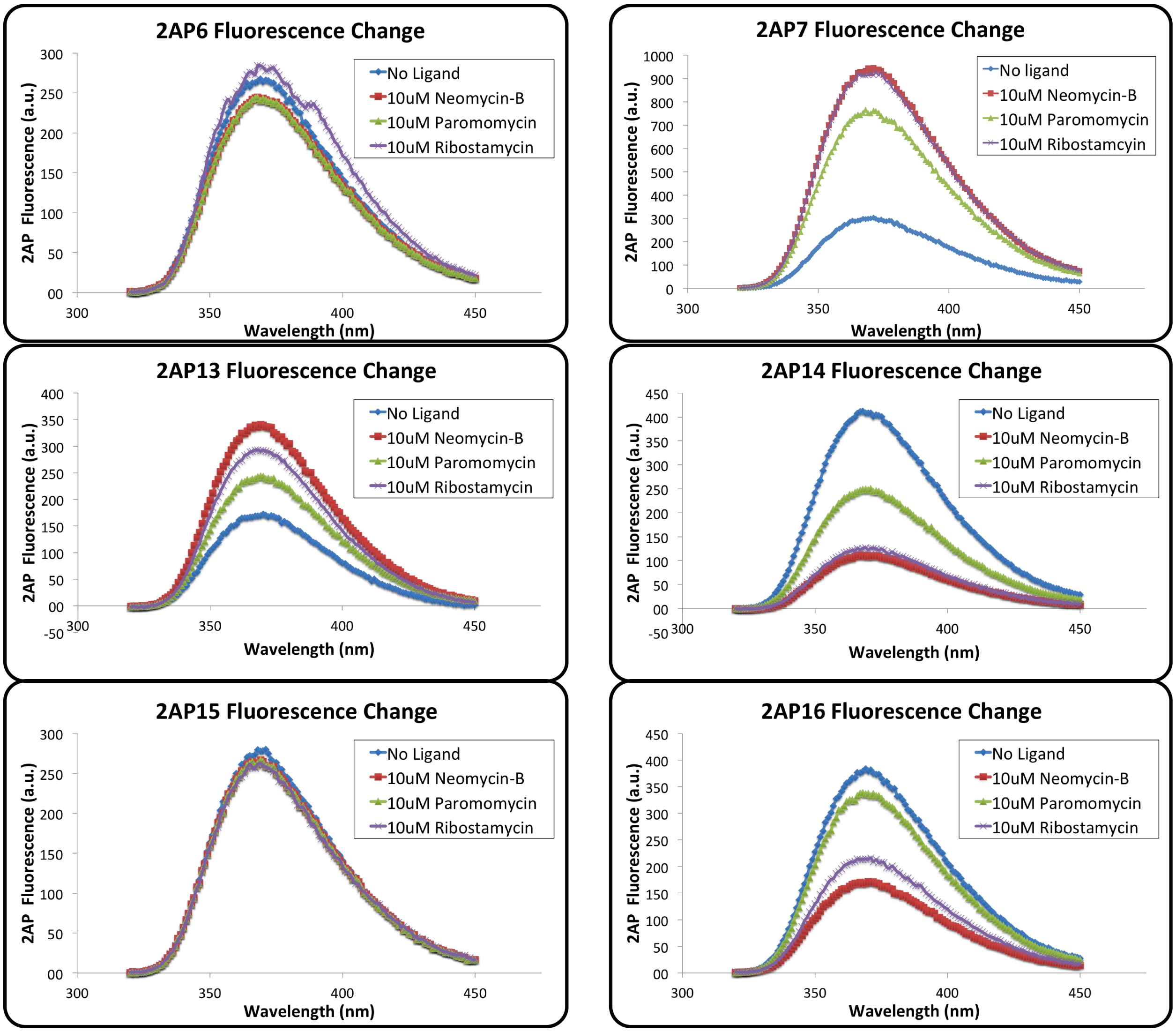

Representative fluorescence scans from 320 nm to 450 nm for 2AP6, 2AP7, 2AP13, 2AP14, 2AP15 and 2AP16 in the presence and absence of ligands after exciting at 307 nm.

Tables

Table S1. Buffers and nucleic acids used in this work

Name	Description
Buffer A	13.5 mM NaCl, 150 mM KCl, 20 mM HEPES, 0.22 mM Na_2HPO_4, 0.44 mM KH_2PO_4, 0.12 mM $MgCl_2$, 120 nM $CaCl_2$, 0.1 mM $MgSO_4$, pH 7.3
Buffer F	80 mM cacodylate, 80 mM KCl, 200 mM NH_4Cl, 5mM $MgCl_2$ at pH 7.3
NEO2A	CAC UGC AGU CCG AAA AGG GCC AGU G
2AP6NEO2A	CAC UG/2AP/ AGU CCG AAA AGG GCC AGU G
2AP7NEO2A	CAC UGC /2AP/GU CCG AAA AGG GCC AGU G
2AP13NEO2A	CAC UGC AGU CCG /2AP/AA AGG GCC AGU G
2AP14NEO2A	CAC UGC AGU CCG A/2AP/A AGG GCC AGU G
2AP15NEO2A	CAC UGC AGU CCG AA/2AP/ AGG GCC AGU G
2AP16NEO2A	CAC UGC AGU CCG AAA /2AP/GG GCC AGU G
NEO2AΔA	CAC UGC AGU CCG AAA GGG CCA GUG

Table S2. Initialization parameters for each molecular dynamics simulation

System	Number of ions added	Number of water molecules	Number of atoms in total
NEO1A unoccupied	22	17006	51784
NEO1A occupied	17	16980	51794

Table S3. Dissociation constants for NEO1A and NEO2A

Ligand	*Kd* (NEO1A) µM (Replicates)	*Kd* (NEO2A) µM (Replicates)
Amikacin	90 (4, 1*)	>100
Geneticin	22.3 ± 9.3 (5)	>100
Kanamycin-A	43 ± 3.6 (3)	26 ± 3.0 (3)
Kanamycin-B	0.75 ± 0.26 (5)	0.25 ± 0.073 (5)
Neomycin-B	0.29 ± 0.054 (8)	0.25 ± 0.050 (8)
Netilmicin	99 (5, 1*)	>100
Paromomycin	1.4 ± 0.19 (5)	1.6 ± 0.26 (5)
Ribostamycin	0.49 ± 0.07 (5)	0.28 ± 0.066 (5)
Sisomicin	8.35 ± 2.9 (3)	4.9 ± 1.2 (3)
Tobramycin	4.8 ± 0.95 (3)	0.40 ± 0.051 (3)

The dissociation constants (K_d) are compared for aminoglycoside binding to NEO1A and NEO2A in Buffer A.

Table S4. Thermodynamic parameters for NEO2A compared with NEO2AΔΔA

Ligand	Aptamer	K_d (μM)	K_d (NEO2AΔΔA)/ K_d (NEO2A)	ΔG (kcal/mol)	ΔH (kcal/mol)	TΔS (kcal/mol)
**Neomycin-B**	NEO2A	0.25 ± 0.05	0.68	-9.0	-9.7	-0.6
	NEO2AΔΔA	0.17 ± 0.031		-9.2	-4.9	4.3
**Paromomycin**	NEO2A	1.6 ± 0.26	N/A	-7.9	-5.8	2.2
	NEO2AΔΔA	No binding				
**Ribostamycin**	NEO2A	0.4 ± 0.075	8.0	-8.9	-11	-2.1
	NEO2AΔΔA	2.3 ± 0.0015		-7.7	-3.7	4
**Sisomicin**	NEO2A	4.9 ± 1.2	1.0	-7.3	-6.1	1.2
	NEO2AΔΔA	4.9 ± 1.0		-7.2	-5.9	1.4
**Tobramycin**	NEO2A	0.40 ± 0.051	15.8	-8.7	-9.1	-0.3
	NEO2AΔΔA	6.3 ± 0.84		-7.1	-2.5	4.6

The dissociation constants (K_d) are compared for aminoglycoside binding to NEO2A and NEO2AΔΔA in Buffer A

References

1. Abraham, M.J.; Murtola, T.; Schulz, R.; Páll, S.; Smith, J.C.; Hess, B.; Lindah, E. Gromacs: High performance molecular simulations through multi-level parallelism from laptops to supercomputers. *SoftwareX* **2015**, *1–2*, 19–25.
2. Lange, O.F.; Van Der Spoel, D.; De Groot, B.L. Scrutinizing molecular mechanics force fields on the submicrosecond timescale with NMR Data. *Biophys. J.* **2010**, *99*, 647–655.
3. Jiang, L.; Majumdar, A.; Hu, W.; Jaishree, T.J.; Xu, W.; Patel, D.J. Saccharide-RNA recognition in a complex formed between neomycin B and an RNA aptamer. *Structure* **1999**, *7*, 817–827.
4. Pettersen, E.F.; Goddard, T.D.; Huang, C.C.; Couch, G.S.; Greenblatt, D.M.; Meng, E.C.; Ferrin, T.E. UCSF Chimera - A visualization system for exploratory research and analysis. *J. Comput. Chem.* **2004**, *25*, 1605–1612.
5. Sousa Da Silva, A.W.; Vranken, W.F. ACPYPE - AnteChamber PYthon Parser interfacE. *BMC Res. Notes* **2012**, *5*, 367.
6. Wang, J.; Wang, W.; Kollman, P.A.; Case, D.A. Automatic atom type and bond type perception in molecular mechanical calculations. *J. Mol. Graph. Model.* **2006**, *25*, 247–260.
7. Wang, J.; Wolf, R.M.; Caldwell, J.W.; Kollman, P.A.; Case, D.A. Development and testing of a general Amber force field. *J. Comput. Chem.* **2004**, *25*, 1157–1174.
8. Jorgensen, W.L.; Chandrasekhar, J.; Madura, J.D.; Impey, R.W.; Klein, M.L. Comparison of simple potential functions for simulating liquid water. *J. Chem. Phys.* **1983**.
9. Essmann, U.; Perera, L.; Berkowitz, M.L.; Darden, T.; Lee, H.; Pedersen, L.G. A smooth particle mesh Ewald method. *J. Chem. Phys.* **1995**, *103*, 8577–8593.
10. Bondi, A. Van der waals volumes and radii. *J. Phys. Chem.* **1964**, *68*, 441–451.
11. A., L. In Molecular Modeling: Principles and Applications. In; Prentice Hall, Harlow, England, 2001; p. 262.
12. Bussi, G.; Donadio, D.; Parrinello, M. Canonical sampling through velocity rescaling. *J. Chem. Phys.* **2007**, *126*.
13. Parrinello, M.; Rahman, A. Polymorphic transitions in single crystals: A new molecular dynamics method. *J. Appl. Phys.* **1981**, *52*, 7182–7190.
14. Hess, B.; Bekker, H.; Berendsen, H.J.C.; Fraaije, J.G.E.M. LINCS: A Linear Constraint Solver for molecular simulations. *J. Comput. Chem.* **1997**, *18*, 1463–1472.
